# Towards Standardized Language to Describe the Pathological Enhancement of the Nipple in NAC-Infiltrating Breast Tumors: A Retrospective Case Series Study

**DOI:** 10.3390/diagnostics15172155

**Published:** 2025-08-26

**Authors:** Cristiana Boldrini, Silvia Amodeo, Angelica Marra, Micol Bottalico, Roberta Dattoli, Riccardo Manfredi

**Affiliations:** Department of Diagnostic Imaging, Oncological Radiotherapy, and Hematology-Diagnostic Imaging Area, Fondazione Policlinico Universitario Agostino Gemelli IRCCS, 00168 Rome, Italyriccardo.manfredi@policlinicogemelli.it (R.M.)

**Keywords:** nipple–areola complex, nipple infiltration, breast tumor infiltrating the NAC, breast MRI

## Abstract

**Background:** The normal pattern of nipple enhancement on magnetic resonance imaging (MRI) is defined based on healthy individuals, as it correlates with the structural anatomy of the nipple–areola complex (NAC). Understanding the normal range of nipple morphology and enhancement on MRI allows radiologists to better identify abnormalities. Some authors have previously detailed the morphology and characteristics of nipple–areola complex enhancement, both in normal and pathologically infiltrating conditions. Our aim is to present a case series involving a population of women with breast cancer infiltrating the NAC, retrospectively evaluated at our institution. Furthermore, based on previously published literature and our own experience, we intend to propose potential standardized language to describe tumor-infiltrating NAC enhancement on MRI and compare it with CT and PET findings. **Methods:** Our study included 110 breast cancer patients with NAC infiltration, who were referred to our hospital from August 2023 to July 2024. All patients were candidates for neoadjuvant chemotherapy and therefore underwent MRI and CT; 33 of them also underwent PET/CT. We distinguished the MRI enhancement pattern based on morphology and intensity. There were three types of morphology: SLE (superficial linear enhancement) at the skin level, NEZ (non-enhancing area immediately below the SLE), and INE (nipple enhancement below the NEZ but above the nipple base). In INE, the pattern could be linear or patchy. Depending on the intensity, the enhancement could be minimal, mild, moderate, or marked. The enhancement on CT depended on the distribution of pathological tissue in the infiltrated NAC and could be present or absent; it could involve the nipple base, the nipple body, or both. For quantitative analysis, we used the maximum standardized uptake value (SUV) measured in early-stage PET/CT images, obtained by delineating a three-dimensional volume of interest (VOI) on the NAC. **Results:** In our population, the most represented enhancement pattern was INE (110), while slightly less than half of the patients showed invasion of the NEZ (49). Approximately one quarter of the patients presented linear ductal INE (36), while the majority presented patchy INE (74). On CT and PET/CT, NAC enhancement was detectable in almost all patients (102), mainly involving the base and the body together. Correlation analysis in the following pairs of variables showed a high association, with a Kendall’s tau value greater than 0.7 (*p* < 0.001): (1) involvement of the NEZ on ce-MR and pattern of nipple involvement on ce-CT (CT score); (2) morphological pattern of INE on ce-MR (INE score) and intensity of INE enhancement on MR; and (3) pattern of nipple involvement on ce-CT (CT score) and intensity of INE enhancement on MR. The calculated mean SUV of pathological NACs on PET/CT for early-stage images was 3.59, while the mean SUV of contralateral normal NACs was 2.12. The calculated mean NAC-SUV ratio was 1.7. **Conclusions:** Although pathological involvement of the NAC cannot always be assessed in the final surgical specimen due to the effects of neoadjuvant chemotherapy, so the “gold standard” of histological reference is missing, MRI and CT with morphology and enhancement descriptors, and additionally PET/CT with SUV measurement can, in our opinion, provide valuable information on the infiltrated nipple. Standardized language for describing breast tumors infiltrating the NAC is desirable to ensure consistent interpretation across different radiologists.

## 1. Introduction

**Figure 1:**
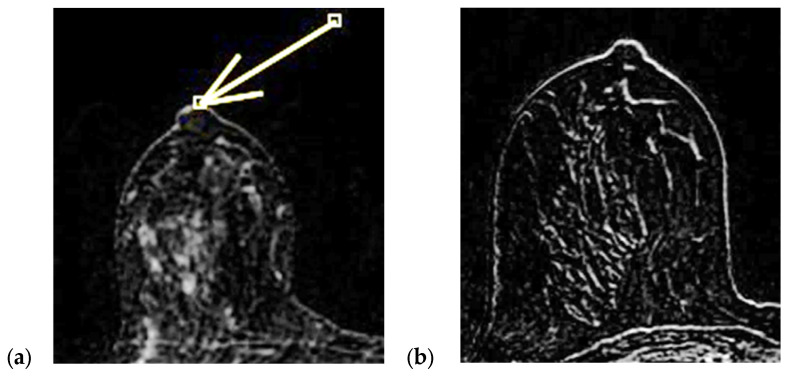
(**a**,**b**) SLE: superficial linear enhancement pattern (smooth superficial enhancement of the NAC at the level of skin) during ce-MR (arrow).

**Figure 2:**
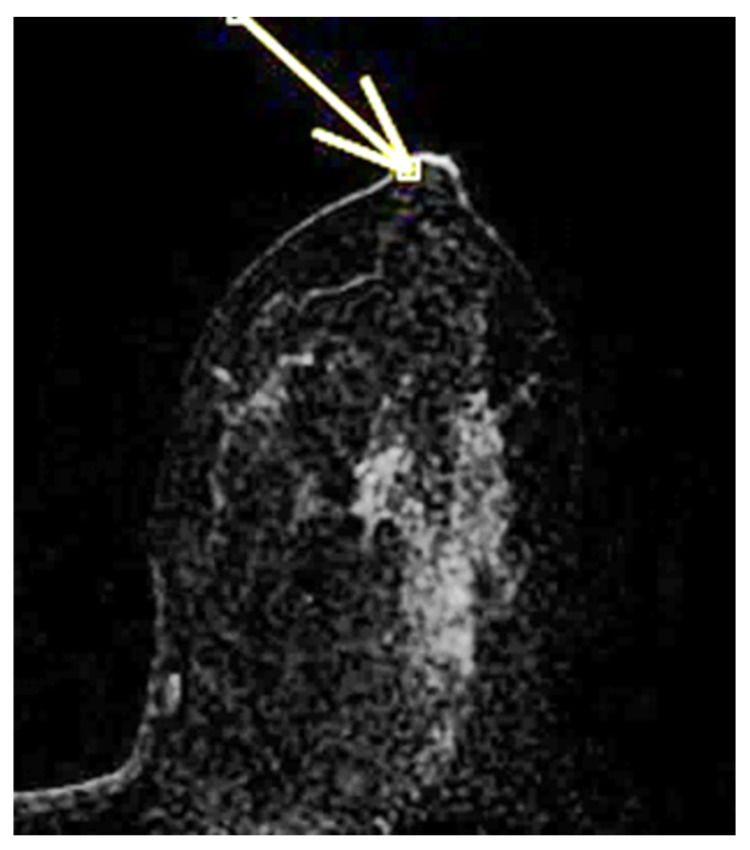
NEZ: non-enhancing zone (zone without enhancement immediately below the SLE) during ce-MR (arrow).

**Figure 3:**
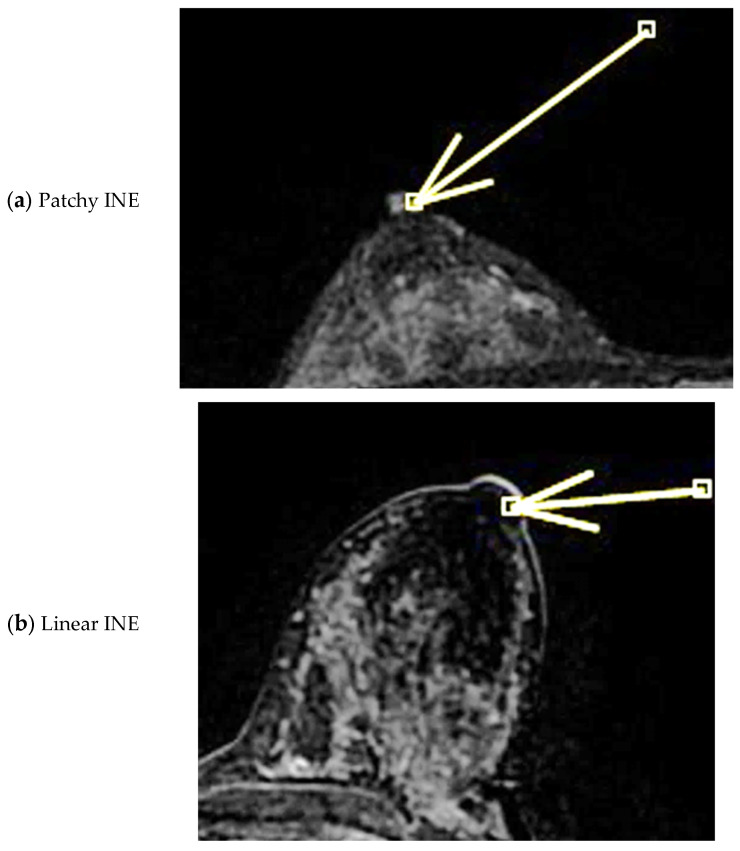
(**a**,**b**) INE: internal nipple enhancement pattern (enhancement of the nipple below the NEZ but above the base of the nipple) during ce-MR. INE can have a patchy (**a**, arrow) or linear (**b**, arrow) morphology.

The physiological pattern of nipple morphology and enhancement on magnetic resonance imaging (MRI) correlates with the normal structural anatomy of the nipple in healthy individuals. Scientific data regarding the assessment of NAC on MRI exist primarily in the context of breast cancer staging and typically distinguish between the presence or absence of nipple–areola involvement without further characterization, especially with regard to morphology and enhancement [1,2,3]. In a paper published in 2019, Gao, Brachtel et al. detailed the concepts of normality and abnormality in the morphology and enhancement characteristics of the nipple–areola complex [4]. The authors distinguished the different morphologies of the normal and pathologically abnormal nipple (everted, flat, or inverted) on MRI and differentiated nipple enhancement patterns based on the distribution of the contrast agent on MRI. In fact, the lack of an objective lexicon in the ACR BIRADS^®^ based on reproducible diagnostic criteria may cause wide variability in interpretations among radiologists worldwide in the evaluation of the NAC. According to the classification proposed by Gao et al., nipple morphology refers to the configuration of the nipple based on its external contour, which is described as everted, flat, or inverted, and is best assessed on MIP MRI images [1,2,4,5,6]. The characteristics and intensity of nipple enhancement can be described using the early findings of post-contrast T1-weighted subtraction MRI [1,2,4,5]. Superficial linear enhancement (SLE) refers to a thin, smooth, superficial rim of enhancement of the NAC at the skin level, with an enhancement intensity greater than that in the immediately adjacent skin (Figure 1) [1,4]. Non-enhancement zone (NEZ) refers to the area without enhancement immediately below the SLE (Figure 2) [1,4]. Internal nipple enhancement (INE) refers to nipple enhancement below the NEZ but above the nipple base, with distinguishable linear or irregular enhancing patterns (Figure 3) [1,4].

Nipple–areola complex (NAC) involvement represents a complex challenge in invasive breast cancer, particularly in selecting patients appropriately indicated for nipple-sparing mastectomy (NSM) for cosmetic reasons [7]. Breast-conserving surgery is commonly performed today, but it is estimated that approximately 30% of patients with invasive breast cancer undergo mastectomy [4]. For women who are candidates for radical surgery, NSM with immediate reconstruction and preservation of the nipple–areola complex and skin is increasing in indications and use, due to patient satisfaction with cosmetic and postoperative outcomes. A 2019 study by Young et al. reported that within 30 days of NSM, the rate of postoperative complications requiring treatment, such as surgical site infection, seroma, hematoma, and necrosis, was only 7.5% in more than 1300 analyzed cases and that this rate further decreased after the learning curve with NSM, even in high-risk patients [8]. Several studies published in the last decade have shown that there is no significant difference in survival outcome between performing NSM and other pre-existing procedures, including skin-sparing mastectomy and conventional total mastectomy [9,10,11,12]. Reports based on histological analyses of mastectomy specimens have suggested that a central tumor location, large tumor size (≥2 cm), nodal positivity, lymphatic vascular invasion (LVI), and multicentricity or multifocality were associated with more likely nipple involvement [5,13]. Traditionally, a tumor-to-nipple distance of at least 2.0 cm has been considered a safe indication for performing NSM. Many recent surgical studies in the last decade have reported that NSM is a feasible treatment option even in patients with a tumor-to-nipple distance less than 2.0 cm [14,15,16,17,18]. New data in the literature (Dent et al., Balci et al.) suggest that a tumor-to-nipple distance (TTN) greater than or equal to (≥) 1 cm but less than or equal to (≤) 2 cm should no longer contraindicate NSM, despite previous contraindications [5,14,19,20]. Usually, the potential for residual occult tumors after NSM may cause some concern in planning the surgery, as nipple preservation is considered a risk factor for recurrence [21]. In 2015, a meta-analysis published by De La Cruz et al. aimed to understand overall survival, disease-free survival, local recurrence, and nipple–areolar recurrence in patients treated with NSM; the authors found that local recurrence rates at NAC were very low [14].

## 2. Materials and Methods

Our study retrospectively evaluated, from August 2023 to July 2024, a population of 110 patients with NAC-infiltrating breast cancer on magnetic resonance imaging (MRI). All patients were candidates for neoadjuvant chemotherapy and therefore underwent breast MRI and whole-body CT before starting chemotherapy; 33 of them also underwent PET/CT to determine skeletal involvement. The mean calculated age was 54.5 years (range 33 to 80 years). This study received approval from our institution’s Ethics Committee, “Comitato Etico Lazio Area 3”, with protocol number ID 6381.

### 2.1. Imaging Techniques and Image Classification

The MRI and CT images were independently analyzed by two different radiologists, with varying degrees of experience in interpreting breast imaging (more than 8 years and more than 3 years, respectively). They recorded lesion characteristics based on a standardized classification (described later in this section), regarding the morphology and intensity of nipple enhancement on MRI and the distribution of nipple enhancement on CT.

Breast MRI was performed on all participants using a 1.5 Tesla MRI unit and a dedicated breast coil. All images were acquired using bilateral axial projections with the patient in the prone position. T1- and T2-weighted turbo spin-echo sequences and T2-weighted fat-suppressed spin-echo sequences were included in the routine protocol. One pre-contrast series and five post-contrast series were included in the dynamic examination, using a T1-weighted gradient-echo sequence with fat suppression. We considered the first post-contrast T1-weighted MR image diagnostic to assess the morphology and intensity of enhancement of infiltrated nipples. The contrast agent administered was Prohance^®^ (gadoteridol, Bracco (Bengaluru, India), 0.1 mmol/kg). For CT, we evaluated only post-contrast scans obtained in the venous phase approximately 60 s after contrast agent injection; scans were acquired using iomeprol, iopromide, or iohexol.

We distinguished the MRI enhancement pattern based on morphology and intensity. Three types of morphology were present: SLE (superficial linear enhancement) at the skin level, NEZ (zone without enhancement immediately below the SLE), and INE (nipple enhancement below the NEZ but above the nipple base). In INE, the pattern could be “linear” or “patchy” (patchy INE with an approximately round shape was more correctly defined as “nodular”). Based on intensity, enhancement was assessed using a subjective qualitative method and could be minimal, mild, moderate, or marked. The enhancement pattern on venous phase CT depended on the distribution of pathological tissue in the infiltrated NAC and could be present or absent; it could involve the nipple base, the nipple body, or both. For quantitative analysis, we used the maximum standardized uptake value (SUV) measured in early-stage PET/CT images (one hour after radioisotope administration), obtained by delineating a three-dimensional volume of interest (VOI) on the NAC. The most representative image was selected, and the VOIs were carefully drawn to avoid adjacent lesion areas. The SUV values for the NAC in the malignant breast and the contralateral normal breast were noted, and we then calculated the NAC-SUV ratio (R) using the following formula: NAC-SUV (R) = SUV of the NAC in the malignant breast/SUV of the NAC in the contralateral normal breast. The NAC-SUV was calculated for early-stage images.

For each patient, tumor histology was determined by ultrasound-guided needle biopsy of the lesion or by definitive histological examination of the surgical specimen.

### 2.2. Statistical Analysis

To perform each statistical correlation between ordinal variables, we used Kendall’s tau (τ-)test.

The term “INE score” refers to the morphological pattern of INE on contrast-enhanced MR (ce-MR), which can be linear, patchy, or nodular. The term “MR score” refers to the pattern of nipple involvement on contrast-enhanced MR (ce-MR), which in our population can be of three different types: involving SLE, NEZ, and INE simultaneously; only INE; or both SLE and INE. The term “CT score” refers to the pattern of nipple involvement on contrast-enhanced CT (ce-CT), which can be nipple base only, nipple body only, or base and body together. Kendall’s tau test was used to determine the correlation between the following pairs of ordinal variables:

1. Morphological pattern of INE on ce-MR (INE score) and tumor grading (G1, G2, and G3);
2. Involvement of the NEZ on ce-MR and pattern of nipple involvement on ce-CT (CT score);
3. Morphological pattern of INE on ce-MR (INE score) and intensity of INE enhancement on MR;
4. Pattern of nipple involvement on ce-CT (CT score) and intensity of INE enhancement on MR;
5. Pattern of nipple involvement on ce-MR (MR score) and pattern of nipple involvement on ce-CT (CT score).

Given the variability in interpretation of the “INE score”, the “MR score”, and the “CT score” between the two differently experienced radiologists who evaluated the images, we calculated Cohen’s kappa coefficient for these three parameters, with the aim of establishing the rate of agreement between the two different evaluating physicians, for each of these three variables.

All statistical analyses were performed with DATAtab (DATAtab 2025: Online Statistics Calculator. DATAtab e.U., Graz, Austria. URL https://datatab.net, accessed on 26 April 2025). Kendall’s tau is a non-parametric rank correlation measure used to assess the strength of the association between two variables. It ranges from −1 to 1; tau values close to 1 indicate a strong association, values close to 0 indicate a weak or no association, and values close to −1 indicate a strong inverse association. Student’s *t*-test was used to determine the statistical significance of the correlations listed in items 1 to 5. *p* Values less than 0.05 were considered statistically significant for the correlation.

## 3. Results and Discussion

### 3.1. Observational Findings: Nipple Morphology, and Nipple Enhancement Pattern on CE-MRI and CE-CT

#### 3.1.1. Nipple Morphology

In our clinical case series study, nipple morphology was most commonly everted (71 cases, 64.5%), less commonly flat (25 cases, 22.8%), and occasionally inverted (14 cases, 12.7%). Areolar and periareolar skin thickening associated with skin and subcutaneous fat enhancement occurred in 40 cases (36.4%) of the population examined.

#### 3.1.2. Nipple Enhancement Pattern on MRI

**Figure 4:**
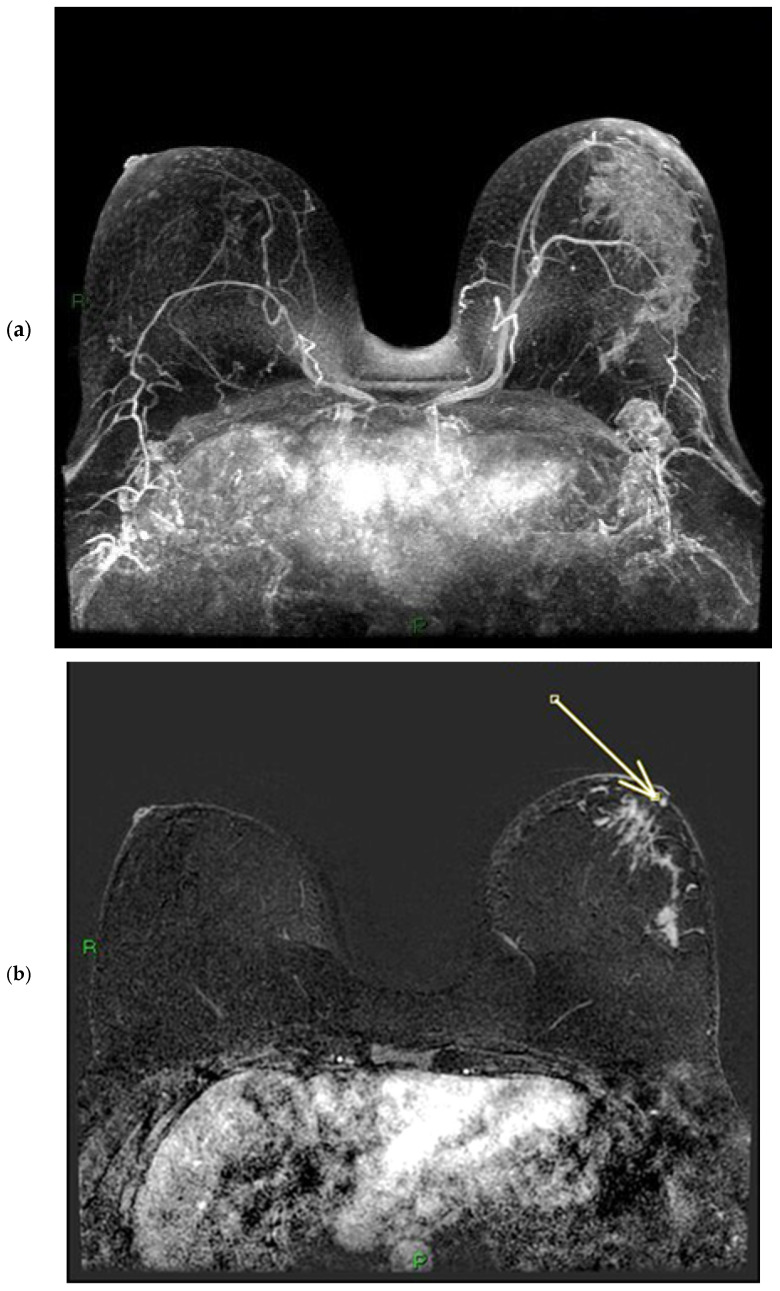
(**a**,**b**) Patient’s name R.M. In the MIP (maximum-intensity projection) reconstruction of the first post-contrast fat-suppressed T1 sequence, we can observe a gross area of pathological enhancement, which affects the external quadrants of the left breast and infiltrates the nipple (**a**). During ce-MR, no SLE and no NEZ are present in the affected left breast nipple; the arrow indicates marked patchy nodular INE of the left breast nipple (**b**).

**Figure 5:**
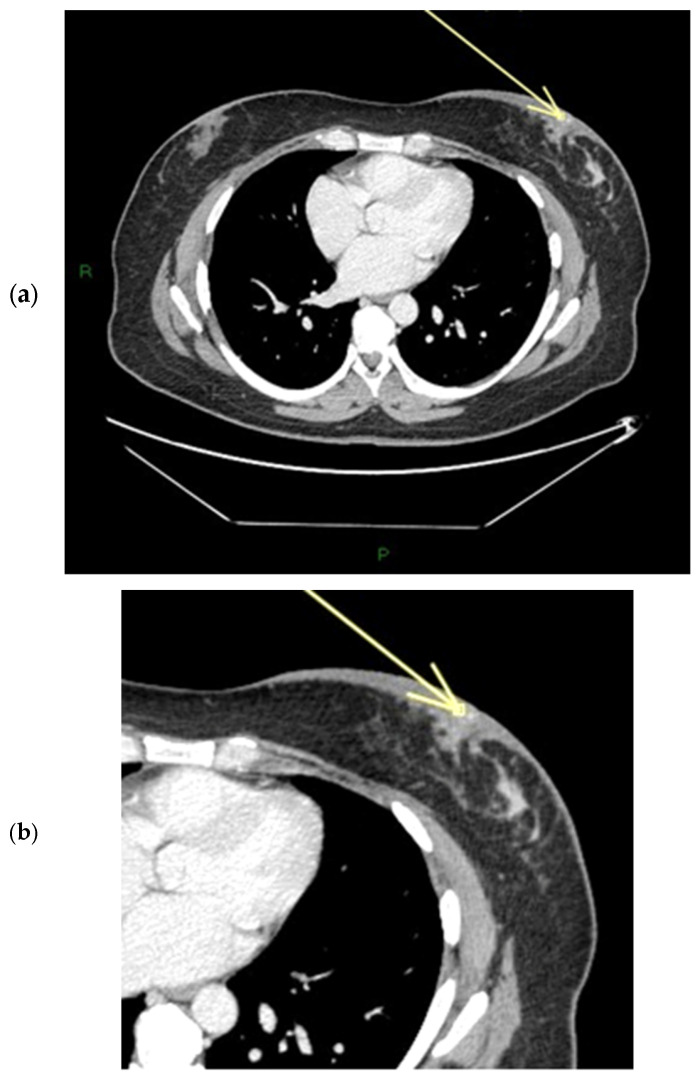
(**a**,**b**) Patient’s name R.M. In the ce-CT scan, the arrow shows enhancement of the base of the left nipple alone (and not of the body).

**Figure 6:**
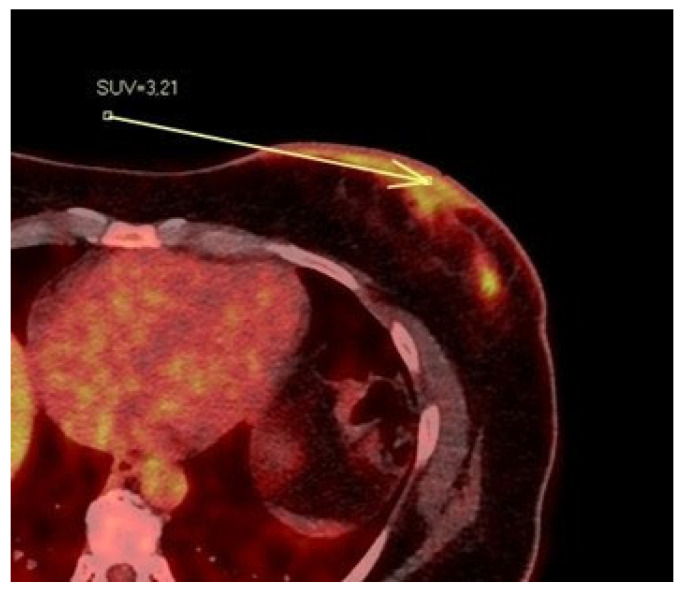
Patient’s name R.M. During PET/CT, the pathological NAC-SUV value is measured at the base of the left nipple.

**Figure 7:**
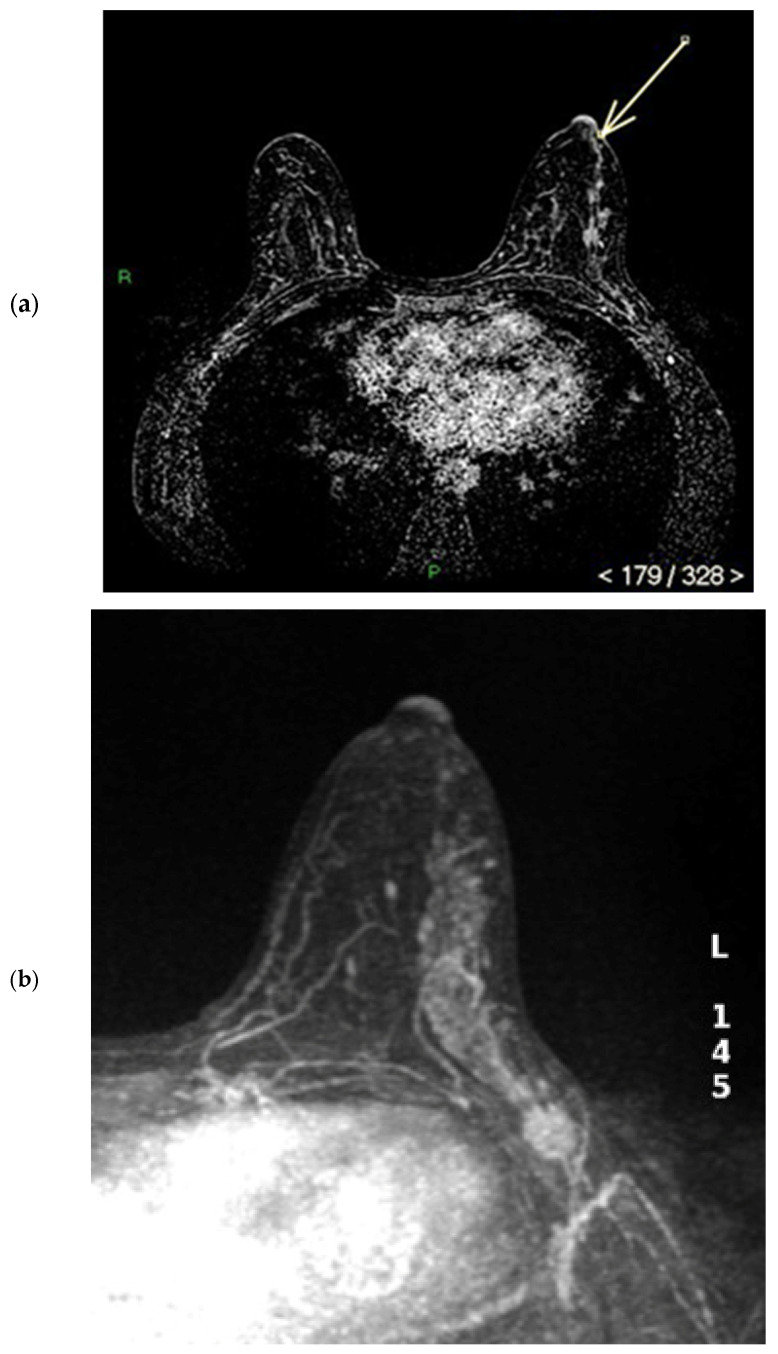

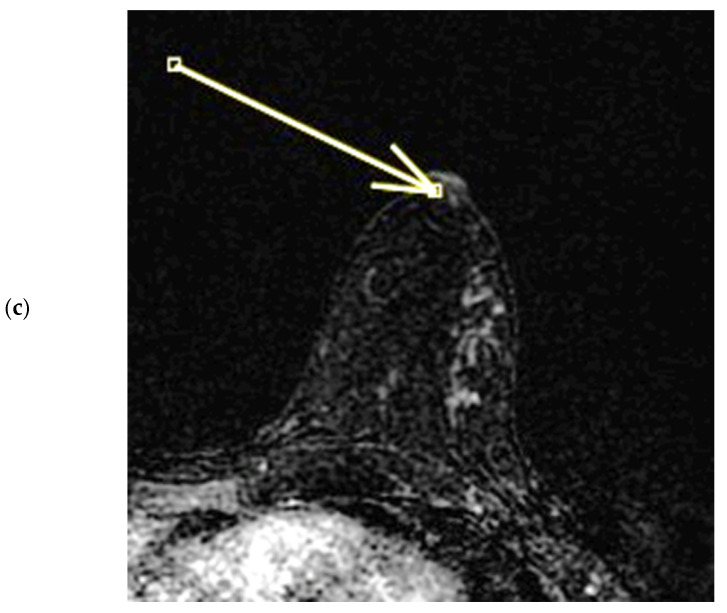
(**a**–**c**) Patient’s name M.E. In the post-contrast fat-suppressed T1 sequences and in the MIP (maximum intensity projection) reconstruction of the first post-contrast fat-suppressed T1 sequence, we notice a gross area of pathological enhancement which affects the external quadrants of the left breast and infiltrates the nipple, without retraction (**a**,**b**). During ce-MR, SLE and NEZ enhancement patterns are present; the arrow indicates mild linear INE of the left breast nipple (**c**).

**Figure 8:**
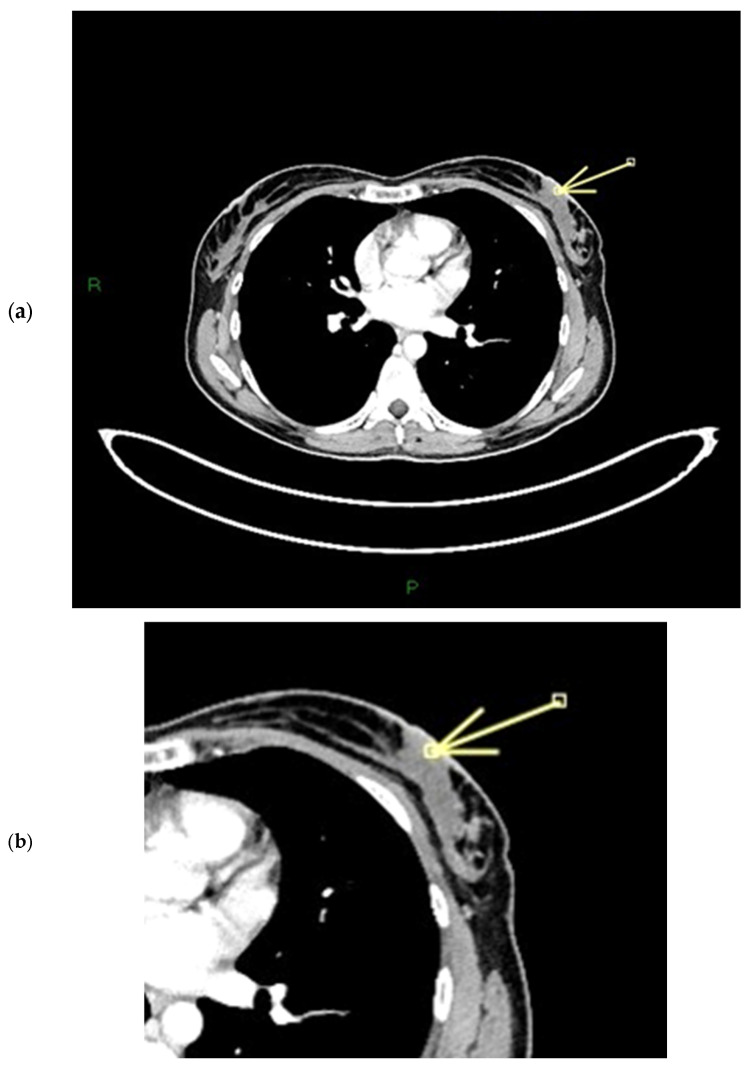
(**a**,**b**) Patient’s name M.E. The ce-CT scan shows enhancement of the base of the left nipple alone (no body; arrow). Morphology of the nipple is everted.

On MRI, pathological nipples most commonly show SLE (90 cases, 81.8%) overlying an NEZ (49 cases, 44.5%), at which the depth of the INE is observed as patchy (74 cases, 67.3%) or linear (36 cases, 32.7%). In 16 cases, the INE is characterized by a “nodular” morphology (14.5%). INE appears to be the most common enhancement pattern in tumor-infiltrated NAC (Figure 4, Figure 5 and Figure 6). When evaluating the kinetics of INE, the majority of infiltrated nipples (53 cases, 48.2%) showed a persistent enhancement pattern, with marked intensity being the most common; 35 patients showed moderate-intensity INE enhancement (31.8%), while 22 patients showed mild-intensity INE enhancement (20%) (Figure 7 and Figure 8). None of the women evaluated showed minimal INE enhancement. The most common INE pattern was patchy (approximately three-quarters of the population, 74 patients), while a linear pattern was observed in only 36 patients.

In 16 cases, INE is characterized by a more specific “nodular” morphology. Among the 16 patients with patchy “nodular” INE, 8 patients have multicentric G3 (high-grade) invasive ductal carcinoma (IDC); this group represents 17.8% of women with the same histological diagnosis (IDC G3—45 patients). Only one of the women with “nodular” INE has a G1 IDC (low-grade invasive ductal carcinoma), while the others have G2—3 IDC (3 patients), G3 ILC (high-grade invasive ductal carcinoma) (2 patients), or G2 ILC (2 patients). If we consider the patients with “patchy” or “nodular” INE (74 of 110), 47 of them have intermediate or high-grade invasive carcinoma. The correlation analysis between morphological pattern of INE on ce-MR (INE score) and tumor grading (G1, G2, G3) is illustrated in the section entitled “statistical correlations”.

#### 3.1.3. Nipple Enhancement Pattern on CT

**Figure 9:**
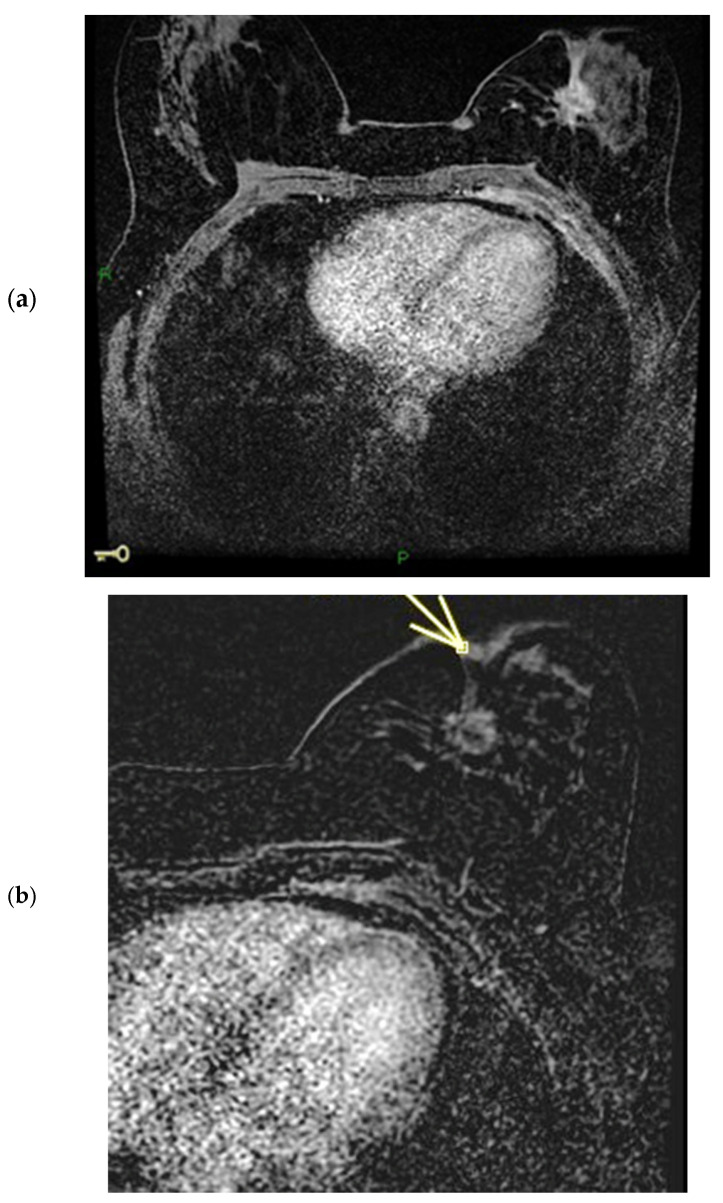
(**a**,**b**) Patient’s name D’A.L. Ce-MR reveals the presence of a gross area of pathological enhancement, which affects the central quadrant of the left breast, with nipple retraction, skin thickening, and ulceration. SLE, NEZ, and moderate patchy INE of the left breast nipple are detected (arrow).

**Figure 10:**
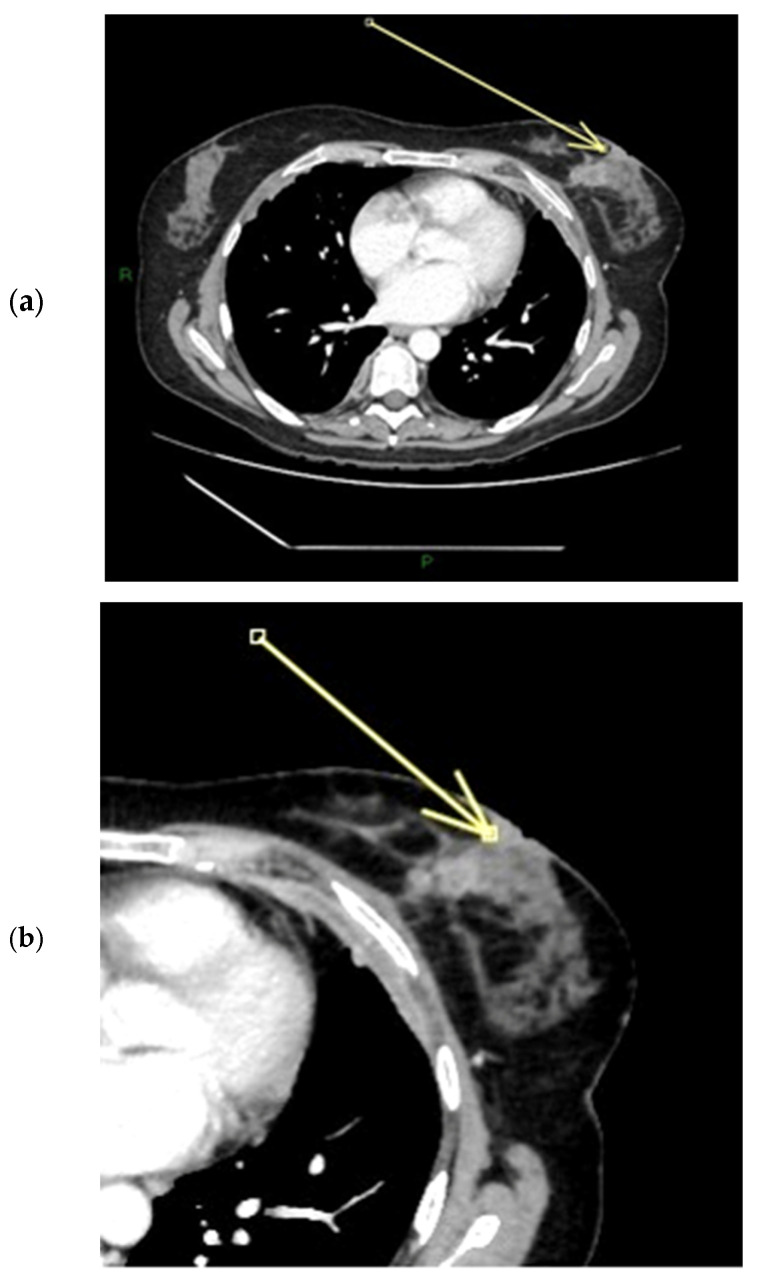
(**a**,**b**) Patient’s name D’A.L. In the ce-CT scan, the arrow shows enhancement of both the base and the body of the left nipple. Morphology is inverted, with skin thickening associated.

**Figure 11:**
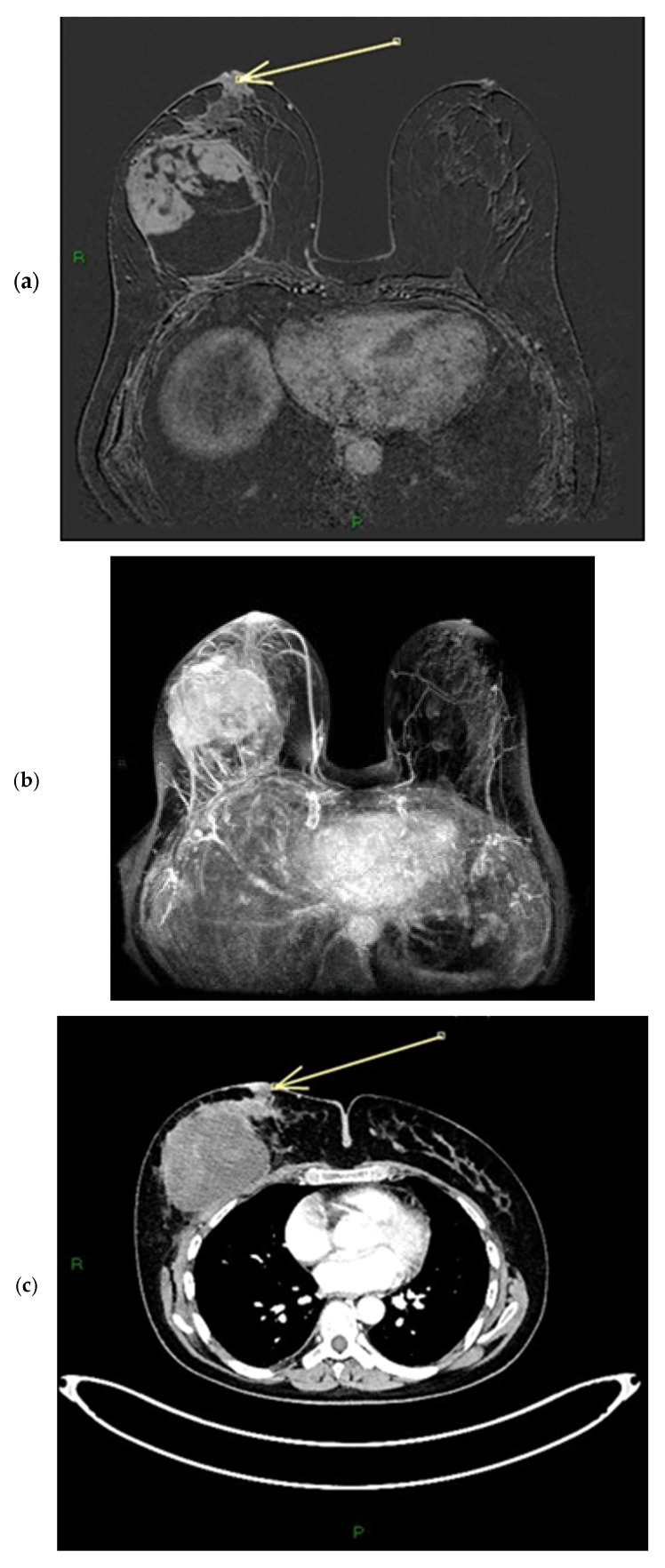
(**a**–**c**) Patient’s name V.F. During ce-MR, we notice a flat thickened right nipple, with the representation of SLE, NEZ, and marked patchy INE pattern of enhancement (**a**). In the ce-T1 sequence (**a**) and in the MIP reconstruction (**b**), we can observe the presence of a partly necrotic mass-like enhancement of at least 6 cm located in the external quadrants of the right breast; the non-mass-like enhancement component that infiltrates the nipple measures approximately 3 cm and extends anteriorly to the gross mass (arrow in **a**). In the ce-CT scan, the arrow shows enhancement of both the base and the body of the right nipple. Morphology is flat, with skin thickening associated (**c**).

**Figure 12:**
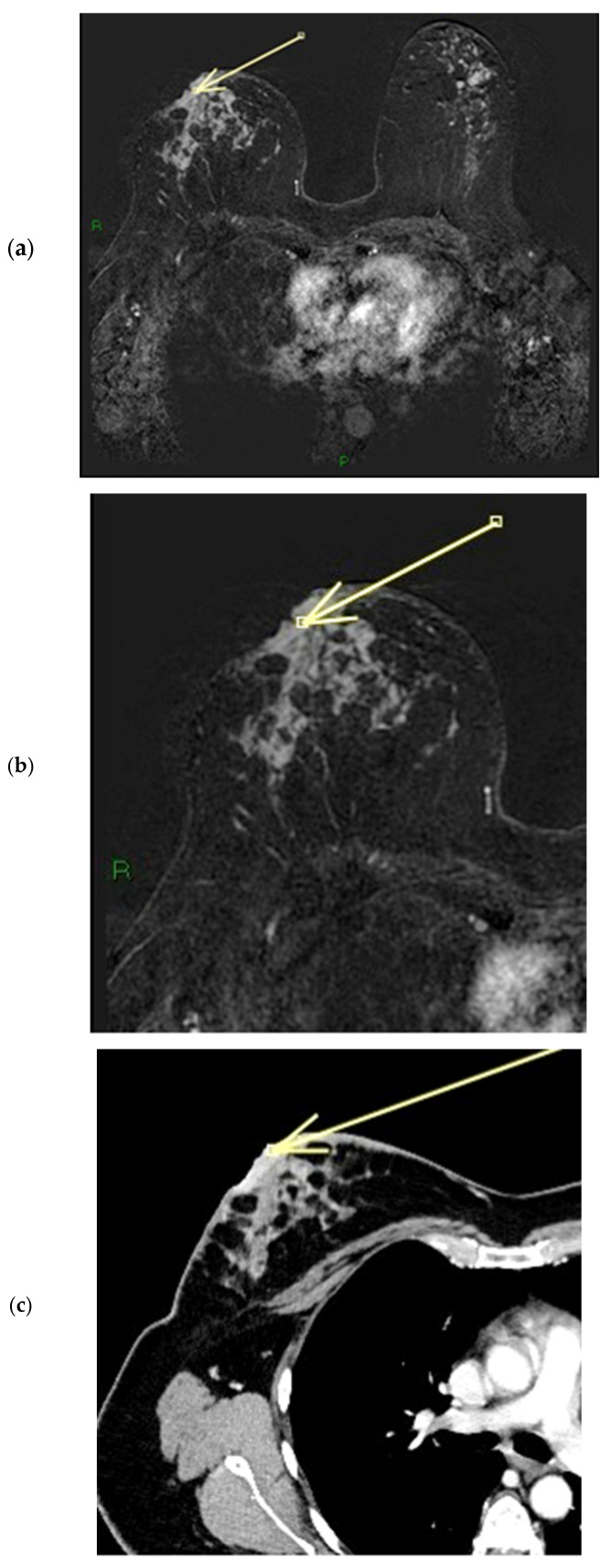
(**a**–**c**) Patient’s name DN.A. The external quadrants of the right breast are occupied by a mass lesion with non-mass peripheral components, which infiltrates the nipple, retracting it. During ce-MR, we see a flat thickened right nipple, with SLE, NEZ, and marked patchy INE pattern of enhancement (arrows in **a**,**b**). In the ce-CT scan, the arrow shows enhancement of both the base and the body of the right nipple. Morphology is inverted, with skin thickening and ulceration (**c**).

Enhancement of the NAC on CT scans acquired in the venous phase was found in 102 cases (92.7% of the population). The most frequent combination of CT enhancement patterns involved the base (base only: 44 cases) and body (body only: 1 case) of the nipple (57 cases, 51.8%) (Figure 9 and Figure 10). When the NEZ is not involved by tumor on MRI (uninvolved NEZ: 61 cases), CT scans usually show enhancement of the base and body together (22 cases, 36%), or of the base of the nipple only (30 cases, 49.2%). In 8 patients, negative NEZ is associated with no enhancement of either the base or the body (13.1%), while only 1 patient with negative NEZ has enhancement of the body of the nipple only (1.6%). The correlation analysis between involvement of the NEZ on ce-MR and pattern of nipple involvement on ce-CT (CT score) is illustrated in the section entitled “statistical correlations”. CT images have a much lower sensitivity than MRI in the detection of SLE; SLE on contrast-enhanced CT was detectable in 23 participants, all patients with marked nipple thickening. This may be due to the lower contrast resolution of CT compared to MRI (Figure 11 and Figure 12).

#### 3.1.4. Histological Findings

Histological findings after needle biopsy are available in 109 of 110 patients; one woman with an infiltrating NAC lesion has an unknown malignant outcome, as the biopsy was performed at another center. The partial distribution of histological findings in 33 of 110 patients (undergoing MRI, CT, and PET/CT) is shown in Table 1. Invasive ductal carcinoma (CDI) was found in 85 patients, with 1 patient having grade G1, 2 patients having G1–G2, 21 patients having G2, 15 patients having G2–G3, the majority (45 patients) having grade G3, and 1 woman with CDI without a specified grade. Invasive lobular carcinoma (ILC) was detected in 15 patients, 8 of whom were grade G2, 1 with grades G2–G3, and 6 with grade G3. One woman had a malignant phyllode tumor (sarcoma); another was diagnosed with angiosarcoma, another with breast ovarian metastasis, and another with invasive mucinous carcinoma; and five women had invasive carcinoma (1 G1–G2, 2 G2, 1 G2–G2, and 1 G3) of unspecified type (neither ductal nor lobular). The observational results for the subgroup of 33 women who underwent MRI, CT, and PET/CT, with details on their histological findings, are summarized in Table 1.

#### 3.1.5. Statistical Correlations

Kendall’s tau test was used to determine the correlation between:

(1) Morphological pattern of INE on ce-MR (INE score) and tumor grading (G1, G2, and G3) (Table 2);
(2) Involvement of the NEZ on ce-MR and pattern of nipple involvement on ce-CT (CT score) (Table 3);
(3) Morphological pattern of INE on ce-MR (INE score) and intensity of INE enhancement on MR (Table 4);
(4) Pattern of nipple involvement on ce-CT (CT score) and intensity of INE enhancement on MR;
(5) Pattern of nipple involvement on ce-MR (MR score) and pattern of nipple involvement on ce-CT (CT score).

The correlations investigated produced the following results:

(1) Kendall’s tau 0.382 (95% CI 0.382–0.382, *p* < 0.001), suggesting a moderate correlation;
(2) Kendall’s tau 0.817 (95% CI 0.817–0.817, *p* < 0.001), suggesting a high correlation;
(3) Kendall’s tau 0.815 (95% CI 0.815–0.815, *p* < 0.001), suggesting a high correlation;
(4) Kendall’s tau 0.710 (95% CI 0.71–0.71, *p* < 0.001), suggesting a high correlation;
(5) Kendall’s tau 0.406 (95% CI 0.406–0.406, *p* < 0.001), suggesting a moderate correlation.

Given the variability in interpretation of the “INE score”, the “MR score”, and the “CT score” between the two different radiologists who evaluated the images, we calculated Cohen’s kappa coefficient for these three parameters. The Cohen’s kappa coefficient value for “INE score” was 0.85, that for “MR score” was 0.9, and that for “CT score” was 0.89, indicating substantially low interobserver variability between the two radiologists who evaluated the images, despite their different degrees of experience in the field.

The patient subgroups evaluated in the correlations at points 1, 2, and 3 are summarized in the following Table 2, Table 3 and Table 4, respectively.

In our population of women, the most common MRI enhancement pattern was INE (100%), with patchy INE being the most common (patchy 67.3%); INE was characterized by a nodular morphology in 14.5% of patients. On CT, enhancement of the NAC was detectable in almost all patients (93%), mainly involving the base and body combined (51.8%). When the NEZ was not involved by tumor on MRI, CT usually showed enhancement of the base and body combined (36%), or of the nipple base alone (49.2%). The correlation analysis between “morphological pattern of INE on ce-MR (INE score) and tumor grading” documented a moderate association between the two variables (tau 0.382), with a high level of significance (*p* < 0.001). Considering the variables as ordinal, the INE score correlates moderately with tumor grading, indicating that grading of breast cancer (G1, G2, and G3) may somewhat influence the morphology of the INE (linear, patchy, or nodular). The correlation analysis between “involvement of the NEZ on ce-MR and pattern of nipple involvement on ce-CT (CT score)” showed a high association between the two variables (tau 0.817), with a high level of significance (*p* < 0.001). Considering the variables as ordinal, the involvement of NEZ on MRI correlates highly with the pattern of NAC enhancement on CT (only base enhancement but no body enhancement, only body enhancement and no base enhancement, or neither base nor body enhancement); this finding may suggest that the nipple infiltration pattern on CT imaging is credible and reliable in its power to predict NEZ involvement on MR imaging.

The correlation analysis between the ordinal variables examined in the following pairs, “morphological pattern of INE on ce-MR (INE score) and intensity of INE enhancement on MR”, “pattern of nipple involvement on ce-CT (CT score) and intensity of INE enhancement on MR”, and “pattern of nipple involvement on ce-MR (MR score) and pattern of nipple involvement on ce-CT (CT score)”, produced different levels of association between the variables evaluated in the pairs (respectively, tau 0.815, 0.710, and 0.406, with *p* < 0.001 in all cases). From the tau values, it is clear that the intensity of INE enhancement on MRI (minimal, mild, moderate, and marked) correlates highly with both the morphological pattern of nipple INE enhancement on MRI (linear, patchy, and nodular) and the distribution pattern of nipple enhancement on CT. The correlation between the “pattern of nipple involvement on ce-MR (MR score)” and the “pattern of nipple involvement on ce-CT (CT score)” is moderate (tau 0.406), suggesting that MRI and CT do not have the same predictive power in describing anatomical nipple involvement and are therefore not superimposable. A sensitivity and specificity study of the individual techniques would be necessary, with the presence or absence of pathological tissue in the postoperative surgical specimen as the “gold standard”. However, this case series study is intended as a descriptive study on a small medium-sized population, not as a large cohort study of the predictive power of individual imaging techniques; to do so, a larger sample size would be needed, and it would also be necessary to have data on post-mastectomy nipple involvement, but this is not always possible as mastectomy occurs after cycles of neoadjuvant therapy, which can cancel out the presence of pathology in the nipple, rendering residual pathology equal to zero. Also, both MRI and CT imaging refer to a staging phase, while the gold standard on the surgical specimen refers to a phase in which neoadjuvant chemotherapy has often determined an almost complete response of the disease; in this way, a time asynchrony is created between the initial instrumental diagnostic phase and the final histological definition of the surgical specimen.

### 3.2. PET/CT Results: NAC-SUV Ratio

In our population, 33 women underwent PET/CT before starting neoadjuvant chemotherapy to assess skeletal status. For each patient, we quantitatively measured the NAC-SUV values in the malignant breast and the contralateral normal breast (Figure 6) in early-stage images (obtained one hour after radioisotope administration), delineating a three-dimensional volume of interest (VOI) over the NAC. The most representative image was selected, and the VOIs were carefully drawn to avoid including adjacent lesion areas, with the exception of the nipple presumed to be infiltrated based on imaging. We calculated the NAC-SUV ratio (R) by considering the ratio of NAC-SUV in the malignant breast to NAC-SUV in the contralateral normal breast. The NAC-SUV R was calculated only for early-stage images. The calculated mean SUV of pathological NACs was 3.59, while the mean SUV of contralateral normal NACs was 2.12. The calculated mean NAC-SUV ratio was 1.7.

Tumor invasion of the nipple is crucial to determine whether the NAC can be preserved; breast MRI is considered the most useful and accurate imaging modality for predicting nipple involvement in patients, to decide whether NSM is safe or not [13,22,23,24,25,26]. Several studies have evaluated nipple–areola complex (NAC) involvement on MRI, based on clinical and pathological features [2,4,8,14]. To our knowledge, some scientific investigations have also been conducted on the efficacy of 18F-fluorodeoxyglucose (FDG) positron emission tomography/computed tomography (PET/CT) for detecting tumor-associated NAC involvement [27,28,29,30,31]. Since nipple symptoms (e.g., nipple erosion, discharge, retraction, and cystic duct dilation) are highly suspicious for NAC invasion, there is a higher possibility of NAC involvement in patients suffering from clinical nipple changes compared to asymptomatic ones. In 2018, Yoo et al. examined 90 patients with invasive breast cancer to demonstrate the predictive value of preoperative prone 18F-FDG PET/CT for determining NAC involvement, by comparing image parameters and clinicopathological factors based on the presence and/or absence of nipple symptoms [27]. SUVmax values for NAC were obtained in the malignant breast and the contralateral normal breast. They calculated the NAC-SUV ratio using the following formula: NAC-SUVratio = SUVmax of NAC in the malignant breast/SUVmax of NAC in the contralateral normal breast. The NAC-SUV ratio was calculated for early (NAC-SUVRearly; one hour after radioisotope administration) and delayed (NAC-SUVRdelay; two hours after radioisotope administration) imaging. Seventeen patients confirmed pathological NAC involvement based on histological findings. After the multivariate analysis, NAC symptoms (*p* = 0.009), tumor multiplicity (*p* = 0.006), NAC-SUVRdelay delay (>1.23, *p* = 0.007), and tumor-NAC distance based on MRI (≤22.0 mm, *p* = 0.003) were found to be independent predictors of NAC involvement. The NAC-SUVRearly PET parameter (*p* = 0.018) showed predictive significance for NAC involvement in the univariate analysis but did not maintain significance after the logistic regression analysis. The authors concluded that delayed 18F-FDG PET/CT is a useful modality for predicting NAC involvement in breast cancer patients, regardless of the presence or absence of NAC symptoms and that delayed PET/CT could be performed to evaluate malignant NAC involvement during the preoperative workup [27].

Although pathological involvement of the NAC cannot always be assessed in the surgical specimen, MRI and PET/CT with SUV measurement can provide valuable information on the tumor-infiltrated nipple. In our population, the mean SUV value of pathological NAC was 3.59, while the mean SUV value of contralateral normal NAC was 2.12. The mean NAC-SUV ratio was 1.7, meaning that pathological NAC has the highest SUV value, approximately one and a half times higher than normal. In our analysis, the mean early NAC-SUV ratio (1.7) measured in early-stage findings is quite close to the late NAC-SUV ratio (1.23) calculated by Yoo et al. [27] in late-stage findings. In our case series study, among the quantitative parameters derived from PET/CT, the early-stage SUV ratio of pathological NAC compared with the contralateral normal breast offers some interesting contribution to determining the involvement of pathological NAC. The early NAC-SUV ratio (1.7) measured by us in early-stage findings (one hour after radioisotope administration) and the late NAC-SUV ratio (1.23) calculated by Yoo et al. [27] in late-stage findings (two hours after radioisotope administration) both represent the level of uptake of radioactive glucose (18F-FDG) by the pathological nipple, albeit at distinct moments of the PET examination. The two parameters appear similar, although not perfectly identical; in the work by Yoo et al. [27], the NAC-SUV ratio was calculated on delayed PET images. However, we believe that the NAC-SUV ratio calculated in our population is informative as to whether PET-CT can provide information on nipple infiltration in breast cancer, considering that the SUV of the infiltrated nipple is on average 1.7 times higher than that of the contralateral healthy nipple.

## 4. Conclusions

A standardized language to describe NAC-infiltrating breast tumors appears to be desirable in order to improve understanding among groups of radiologists. Gao, Brachtel et al. in 2019 [2] detailed the morphology and characteristics of nipple–areola complex enhancement. Based on the published literature and our retrospective case series study experience, in Table 5, we provide a summary of standard descriptors of nipple morphology and intensity on MRI and the distribution of nipple enhancement on CT. The main limitations of this study are the small number of patients involved and the small number of patients who underwent PET-CT (33 women out of a total of 110 recruited patients), which would make it useful to consider an expansion of the case series, in order to make the collected data and the statistical results more relevant and significant. A sensitivity and specificity study of the individual techniques (MR, CT, and PET) would be necessary, with the presence or absence of pathological tissue in the postoperative surgical specimen as the “gold standard”. This case series study is mainly intended as a descriptive study of “imaging parameters” on a small medium-sized population, not as a study of the predictive power of individual imaging techniques. To do this, a larger sample size would be needed, and it would also be necessary to have definitive histological data on post-mastectomy nipple involvement. Furthermore, we must remember two critical issues that must be addressed in a sensitivity–specificity study: the first is that neoadjuvant therapy cycles can render the pathological residue equal to zero, and the second is that both MRI and CT imaging refer to a staging phase, while the gold standard on the surgical specimen refers to a phase in which neoadjuvant chemotherapy has often determined an almost complete response of the disease. In this way, a time asynchrony is created between the diagnostic phase and the final histological definition of the surgical specimen.

## Figures and Tables

**Table 1:** Radiologic correlation between MR and CT findings in the subgroup of 33 patients who underwent PET/CT.

Name		NAC Enhancement MR		INE Pattern	Intensity MR	NAC Enhancement CT	Base (CT)	Body (CT)	SUV PET 1 (Involved NAC)	SUV PET 2 (Control)	RATIO	Histology
	SLE	NEZ	INE									
D’A.L.	yes	yes	yes	patchy	moderate	yes	yes	yes	3.20	1.47	2.18	IDC G2
L.R.	no	no	yes	patchy	marked	yes	yes	yes	3.89	2.51	1.55	ILC G2
S.E.	yes	yes	yes	patchy	marked	yes	yes	yes	2.06	1.53	1.35	IDC G2
G.N.	no	no	yes	patchy	moderate	no	no	no	2.91	2.18	1.33	ILC G2
A.R.	Yes	no	yes	patchy	moderate	yes	yes	yes	4.41	3.12	1.41	IDC G1-G2
G.EC.	no	no	yes	patchy	marked	yes	yes	yes	6.32	3.20	1.98	IDC G3
C.D.	yes	yes	yes	patchy	marked	yes	yes	yes	7.45	3.04	2.45	IDC G3
B.N.	yes	yes	yes	patchy-nodular	marked	yes	yes	yes	2.10	1.47	1.43	IDC G3
DN.A.	yes	yes	yes	patchy	marked	yes	yes	yes	9.06	3.05	2.97	ILC G2
Af.Re.	no	no	yes	patchy	moderate	yes	yes	yes	2.19	1.81	1.21	IDC G3
V.F.	yes	yes	yes	patchy	marked	yes	yes	yes	2.65	2.01	1.31	Sarcoma
S.G.	no	no	yes	linear	moderate	yes	yes	no	2.00	1.19	1.68	IDC G2
M.E.	yes	yes	yes	linear	mild	yes	yes	no	2.26	2.02	1.12	IDC G2-G3
R.M.	no	no	yes	patchy-nodular	marked	yes	yes	no	3.21	2.91	1.10	IDC G3
P.A.	yes	yes	yes	patchy	marked	yes	yes	yes	6.57	2.22	2.96	angiosarcoma
C.A.	yes	no	yes	linear	moderate	yes	yes	no	4.77	2.17	2.20	IDC G2
DS.R.	no	no	yes	linear	mild	yes	yes	no	2.69	2.08	1.29	IDC G2
DS.C.	yes	yes	yes	patchy	mild	yes	yes	no	2.04	1.82	1.12	IDC G3
DV.V.	yes	no	yes	patchy-nodular	marked	yes	yes	no	2.68	1.77	1.51	IDC G3
A.S.	no	no	yes	linear	moderate	yes	yes	no	2.64	1.72	1.53	IDC G3
S.D.	yes	no	yes	linear	moderate	yes	yes	no	3.57	1.82	1.96	IDC G2-G3
DG.D.	yes	no	yes	linear	marked	yes	yes	yes	3.69	1.91	1.93	IDC G2
B.G.	yes	yes	yes	patchy	marked	yes	yes	yes	3.89	2.77	1.40	IDC G3
G.L.	yes	yes	yes	patchy	marked	yes	yes	yes	2.79	1.78	1.57	IDC G3
Gu.Li.	yes	no	yes	linear	moderate	yes	yes	no	3.82	2.53	1.50	IDC G3
DI.MC.	yes	no	yes	patchy	moderate	yes	yes	no	3.10	2.46	1.26	ILC G3
T.E.	yes	yes	yes	patchy	marked	yes	yes	yes	2.56	1.92	1.33	IDC G2
P.I.	yes	no	yes	linear	moderate	yes	yes	no	4.05	2.36	1.72	IDC G2
N.I.	yes	no	yes	linear	mild	no	no	no	2.92	2.05	1.42	IDC G3
B.D.	yes	no	yes	linear	mild	no	no	no	1.87	1.07	1.74	IDC G3
O.L.	yes	no	yes	patchy-nodular	marked	yes	yes	yes	4.14	1.27	3.26	ILC G3
M.T.	yes	yes	yes	patchy	marked	yes	yes	yes	3.57	2.15	1.66	ICD G3
P.L.	yes	no	yes	linear	moderate	no	no	no	3.52	2.55	1.38	ILC G2

**Table 2:** The table summarizes the number of patients for each subgroup evaluated in the “INE score—tumor grading” correlation.

	Histology G1	Histology G2	Histology G3
Linear INE (36)	1	13	22
Patchy INE (58)	1	23	34
Nodular INE (16)	1	2	13

**Table 3:** The table summarizes the number of patients for each subgroup evaluated in the “NEZ involvement—CT score” correlation.

	Involved NEZ (MR)	Not Involved NEZ (MR)
Involved NAC + BASE + BODY (CT)	35	0
Involved NAC + BASE (no body)	14	30
Involved NAC + BODY (no base)	0	1
No involvement of NAC, body, base	0	8

**Table 4:** The table summarizes the number of patients for each subgroup evaluated in the “INE morphology score—enhancement intensity of INE” correlation.

	Minimal Enhancement (0)	Mild Enhancement (22)	Moderate Enhancement (35)	Marked Enhancement (53)
Linear INE (36)	0	15	18	3
Patchy INE (58)	0	6	16	36
Nodular INE (16)	0	1	1	14

**Table 5:** Standard descriptors of morphology and intensity of nipple–areola complex (NAC) enhancement during MR and distribution of nipple enhancement during CT.

	Magnetic Resonance	CT
**Involvement**	Yes/No	Yes/No
**Pattern of Distribution**	SLE NEZ INE (linear, patchy, nodular)	Base Body Base + Body
**Intensity of Enhancement**	Minimal Mild Moderate Marked	/
